# Gandouling Tablets Inhibit Excessive Mitophagy in Toxic Milk (TX) Model Mouse of Wilson Disease via Pink1/Parkin Pathway

**DOI:** 10.1155/2020/3183714

**Published:** 2020-12-14

**Authors:** Jing Zhang, Lu-Lu Tang, Liang-Yong Li, Shen-Wei Cui, Shan Jin, Huai-Zhen Chen, Wen-Ming Yang, Dao-Jun Xie, Gu-Ran Yu

**Affiliations:** ^1^Department of Neurology, The Affiliated Hospital of Nanjing University of Chinese Medicine, Nanjing, China; ^2^Department of Neurology, First Affiliated Hospital of Anhui University of Traditional Chinese Medicine, Hefei, China

## Abstract

**Objective:**

Gandouling (GDL) tablet is a Chinese patent medicine approved by the National Medical Product Administration, which is used to treat Wilson disease (WD) in China. In this study, we aimed to investigate the effects of GDL on mitophagy in the hippocampus in the toxic milk (TX) mouse model of WD.

**Methods:**

Mice were randomly divided into the following four groups: control, Wilson (model group), D-penicillamine (DPA), and GDL groups. The animal behaviors were evaluated by the water maze experiment, traction test, and pole test. Transmission electron microscopy was used for the detection of mitochondrion structure. An enzyme-linked immunosorbent assay (ELISA) was performed for the analysis of the changes in liver function. Colocalization of mitophagy-related proteins was detected by fluorescence microscopy. Western blotting (WB) and reverse transcription-polymerase chain reaction (RT-PCR) were conducted for the detection of protein expression and mRNA levels, respectively.

**Results:**

Significant reduction in neurological impairments was observed in the WD model group. All of these results were significantly reversed by GDL intervention. Compared with the levels in the Wilson group, the levels of alanine aminotransferase (ALT), aspartate transaminase (AST), total bilirubin (TBIL), and albumin (ALB) changed obviously. Colocalization between mitophagy-related proteins pink1, parkin, and mitochondria was changed significantly. The mitophagy-related mRNA (pink1, parkin, and LC3II) and protein expression levels (pink1, parkin, and the rate of LC3II/LC3I) were decreased significantly, while p62 was remarkably increased after GDL intervention.

**Conclusion:**

Our findings indicated that the neuroprotective mechanism of GDL may occur via the inhibition of excessive mitophagy through the regulation of the pink1/parkin pathway in the TX mouse brain of WD.

## 1. Introduction

Wilson's disease (WD) is an inborn error of copper metabolism, which is caused by autonomic recessive inheritance. This rare disease is caused by mutations in the gene encoding a copper-transporting P-type ATPase (ATP7B), which is critical for copper excretion in the bile, resulting in copper accumulation, mainly in the liver, brain, cornea, and kidney [1]. WD is lethal if left untreated, but it has also been reported to be one of the rare inherited diseases for which effective pharmacologic treatment can be applied. Early diagnosis and timely treatment contribute to effective control, whereas poor prognosis contributes to the life-threatening situation [2].

Copper has been reported to be an essential trace element, which provides a stabilizing feature for chromatin and forms redox-active centers for multiple metalloproteins [3, 4]^.^ However, excessive inorganic copper induces oxidative stress and leads to multisystem and multiorgan dysfunction [5–7]. A recent study has shown that apoptosis, autophagy, and mitochondrial dysfunction are involved in excessive copper induced-oxidative stress damage in male germ cells [8].

Mitophagy is considered to be a selective process of removing damaged mitochondria, which can maintain the balance between mitochondrial quantity and quality [9]. Enhanced mitophagy has been reported to attenuate mitochondrial dysfunction after neuronal degeneration [10, 11]. However, deleterious autophagic processes could lead to cell death, and attenuated mitophagy may protect the neurons from cerebral injury [12]. It is generally believed that the failure of adequate removal of damaged mitochondria or excessive degradation will lead to cell death [13, 14]. To date, some studies have shown that excessive inorganic copper induces oxidative stress and mitophagy, which can lead to mitochondria dysfunction and then aggravate cell apoptosis [8, 15, 16]. However, the role of mitophagy in WD has remained obscure. A recent study has reported that mitochondria in ATP7B-deficient cells was preferentially subjected to autophagy in Wilson disease [17]. Therefore, the regulation of mitochondria, especially in mitophagy, would be a new therapeutic approach for WD.

Although therapeutic approaches for WD have shown significant advancement, the options for pharmacological treatments are still limited, especially for WD patients with neurological symptoms [18, 19]. *D*-penicillamine (DPA), a copper chelator, is the currently available first-line therapy for WD. Although the clinical benefits of treatment with DPA have been documented in detail, the serious side effects, reported up to 50% of patients with neurological WD, have vastly limited its widespread application [20, 21]. Traditional Chinese medicine (TCM) offers opportunities to treat WD-associated neurological impairment, and it has been widely used in China for this purpose. Gandouling tablet (GDL), a Chinese patent medicine, was approved to treat WD in China for several decades (Z20050071). GDL is made up of *Rheum officinale* Baill. (Dahuang*), Coptis chinensis* Franch. (Huanglian)*, Scutellaria baicalensis* Georgi (Huangqin), *Curcuma longa* L. (Jianghuang), *Curcuma rcenyujin Y* (Yujin), *Salvia miltiorrhiza* Bge. (Danshen), and *Curcuma phaeocaulis* Valeton (Ezhu). To date, significant improvement in neurological function among WD patients has already been confirmed by GDL treatment in our previous studies [22, 23]. However, the underlying mechanism of GDL in WD is still unclear. Therefore, it is meaningful and valuable to investigate the mechanism of underlying GDL effects in WD.

At present, comprehensive studies can rarely be performed on human beings due to medical ethics. Therefore, the use of an animal model would be necessary for our further study. The toxic milk (TX) mouse is an ideal animal model for WD, which is considered to be a spontaneous mutation in the orthologous murine gene ATB7B [24]. In our previous study, the effect of alleviating cell apoptosis in TX mice on WD has been confirmed by GDL intervention [25]. However, whether GDL could regulate mitophagy and thus attenuate brain injury in WD has not yet been studied.

Currently, it is known that the Pink1/parkin mitophagy pathway plays a critical role in the autophagic removal of damaged mitochondria, and it has also been reported to be involved in WD [17]. In this paper, we aimed to explore the effects of GDL treatment on mitophagy and mitochondrial function through the Pink1/parkin pathway in the TX mouse model of WD.

## 2. Methods and Materials

### 2.1. Ethics Statement

Thirty-six male (age: 4 months) TX mice (30 ± 5 g) and 12 inbred strain normal mice (DL mice) were obtained from the Anhui Experimental Animal Centre. All mouse experiments in this study were performed at the Animal Experimental Centre of Anhui University of Chinese traditional medicine. The application of animal protocol was reviewed and approved by the Animal Ethics Committee of the Affiliated Hospital of Anhui University of Chinese traditional medicine (2018AH-24). After intraperitoneal injection of sodium pentobarbital (50 mg/kg), cervical dislocation was performed to sacrifice the animals.

### 2.2. Establishment of Animal Models

A total of 36 male (age: 4 months) TX mice were randomly divided into the following 3 groups: Wilson group, GDL group, and DPA group, 12 mice in each group. GDL was produced by Anhui Hospital of Traditional Chinese Medicine. The active ingredients of each herb were extracted with 65% ethanol and then combined with the extracted filtrate. The filtrate compound was baked into dry paste at the right temperature, and starch was added and packed into the GDL troche.

A total of 12 male DL mice (30 ± 5 g) from the animal experimental center in Anhui were treated as the control group. Mice in the GDL group were given GDL by intragastric administration at a dose of 0.486 g/kg/d, while mice in the DPA group were given DPA by intragastric administration at a dose of 0.09 g/kg/d, respectively. Drug administrations were performed twice a day for 8 weeks. Other mice in the Wilson group and control group were given the same volume of distilled water by intragastric administration. All mice were housed in a humidity-controlled room at 50–70% and a temperature-controlled room at 18–22°C with an alternating 12 h light/dark cycle. These animals were fed in an isolated cage, and they were allowed to move and feed freely.

### 2.3. Behavioral Testing

#### 2.3.1. Morris Water Maze Test (MWM)

To investigate spatial learning and memory in all mice, the MWM test was initiated after 8 weeks in the Xinan Medicine Center of Anhui University of Chinese traditional medicine, as previously described by Rechard G. Morris. Each mouse was trained twice a day for a 4-day training, and each interval time exceeded 15 minutes. The mouse was monitored until it reached the platform. When the mouse did not reach the platform in 60 seconds, the experimenter guided it to the platform, as during training. Either way, the animal was allowed to rest for 10 seconds, and then, it was dried off and returned to a holding cage. The mouse was monitored until it reached the platform, and then the times traveled in the last trial were recorded and calculated. After all animals completed the trial, they performed one probe trial, in which the platform was removed from the pool. The probe trial was performed to verify the animal's understanding of the platform location, and we observed the strategy that the animal follows when it discovered the platform was not there. Then, the number of times the animal crossed the center of the pool during 120 seconds was recorded [26]. A digital camera was mounted above the water maze to capture images, and then the images were transmitted to smart software in a personal computer.

#### 2.3.2. Traction Test

A traction test was used to observe the neurological deficits in all mice. The two forelimbs of mice were suspended on a horizontal wire. Traction test scores were graded as follows: 3, mice grasped the wire with two hind limbs; 2, mice grasped the wire with one hind limb; 1, mice could not grasp the wire with both hind limbs [27].

#### 2.3.3. Pole Test

The pole test was also performed as described by Ohno [28]. Briefly, mice were placed with their heads upward at the top of a wooden pole (diameter: 8 mm, height: 45 cm), and then the time periods required for the animal to completely rotate downwards (*T*_turn_) and to descend to the floor were recorded (*T*_total_). Training (3–5 min/session/day) for 3 days in the pole-descending behavior was received by all animals. The prolongation of *T*_turn_ or *T*_total_ was used in the evaluation of neurological deficits and bradykinesia.

### 2.4. Transmission Electron Microscopy

The hippocampus was cut into 1 mm^3^ cubes, immersed in 2.5% glutaraldehyde at 4°C, and immobilized in 1% osmium tetroxide for 2 hours. The specimens were dehydrated in acetone of various graded series and then soaked and embedded. Further, 60 nm ultrathin sections were cut and double-stained with lead citrate and uranyl acetate. The morphology of mitochondria and mitophagy was photographed using a transmission electron microscope (JEM-1400, Tokyo Japan).

### 2.5. Enzyme-Linked Immunosorbent Assay (ELISA)

The blood sample was collected from the abdominal aorta. The supernatant was measured with alanine aminotransferase (ALT), aspartate transaminase (AST), Total bilirubin (TBIL), and albumin (ALB) by ELISA kits (Nanjing Jian Cheng Bioengineering Institute, Nanjing, China), according to the protocols of the manufacturer. The concentration was calculated according to the corresponding optical density (OD) value.

### 2.6. Immunofluorescence

The hippocampus sections were washed using phosphate-buffered saline (PBS : PH 7.4), after being dewaxed by the antigen repair method (EDTA : PH 9.0). After that, they were preincubated in 5% BSA in phosphate buffer for 30 minutes, and then they were incubated overnight using primary antibodies for parkin and p62 (1 : 100; Abcam, Shanghai, China) at 4°C overnight and secondary antibodies (1 : 100; Abcam, Shanghai, China) for 1 h, respectively. Finally, the sections were mounted with mounting medium (containing DAPI; Beyotime, Shanghai, China) and photographed with an epifluorescence microscope (Nikon, Tokyo, Japan).

### 2.7. Real-Time Quantitative Polymerase Chain Reaction (RT-PCR) Analysis

Total RNA samples were extracted from the right hippocampus tissues using EZ-10 Total RNA Mini-Preps Kit Reagent (Sangon Biotech, Shanghai, China). The concentration and purity of total RNA were detected by Ultraviolet spectrophotometry (Jinghua, Shanghai, China). The synthesis of the first-strand cDNA was performed by the First Strand cDNA Synthesis Kit, according to the protocol of the manufacturer (ABclonal, Wuhan, China). The real-time PCR system was used for the detection of the quantitative RT-PCR (Bio-Rad, Shanghai, China). The 2^−ΔΔCt^ method was used for the relative expression level of each gene based on the reference expression of *β*-actin. The primer sequences used for PCR were as follows: parkin forward primer: 5′-GAGGTCGATTCTGACACCAGC-3'; parkin reverse primer: 5′-CCGGCAAAAA TCACACGCAG-3'; pink1 forward primer: 5′-TTCTTCCGCCAGTCGGTAG-3'; pink1 reverse primer: 5′-CTGCTTCTCCTCGATCAGCC-3'; LC3II forward primer: 5′-TTATAGAGCGATACAAGGGGGAG-3'; LC3II reverse primer: 5′-CGCCGTC TGATTATCTTGATGAG-3'; p62 forward primer: 5′-CATCGGAGGATCCGAGTG TG-3'; p62 reverse primer: 5′-TTCTTTTCCCTCCGTGCTCC-3'; beta-Actin forward primer: 5′-GGGAAAT CGTGCGTGACATTAAGG-3'; beta-Actin reverse primer: 5′-CAGGAAGGAAGGC TGGAAGAGTG-3'.

### 2.8. Western Blotting

The protein was extracted from hippocampus tissues using RIPA lysis buffer (1 : 100; Beyotim, Shanghai, China). Equal amounts of protein were separated using the SDS-PAGE Gel Preparation Kit (Sangon Biotech, Shanghai, China). Electrophoresis was carried out in the SDS-polyacrylamide gel using 20 *μ*g of total protein in each lane. After electrophoresis, the protein was transferred to a PVDF membrane. Then, the membrane was incubated with corresponding primary antibodies (pink1 1 : 1000; Abcam, Shanghai, China) at 4°C overnight and the secondary antibody (1 : 10000; Abcam, Shanghai, China) for 1 hour, respectively. Eventually, the targeted antigens were detected by standard chemical luminescence methods. Band intensities were measured with Image software.

### 2.9. Statistical Analysis

Quantitative data were expressed as the mean ± SD and analyzed with SPSS 22.0 software (International Business Machines Corporation, Armonk, NY, USA). All data were analyzed using one-way analysis of variance, followed by the least significant difference test or Tamhane's T2 test. *AP* value < 0.05 was considered statistically significant.

## 3. Results

### 3.1. Effects of GDL on the Behavior of TX Mice with WD

**Figure 1:**
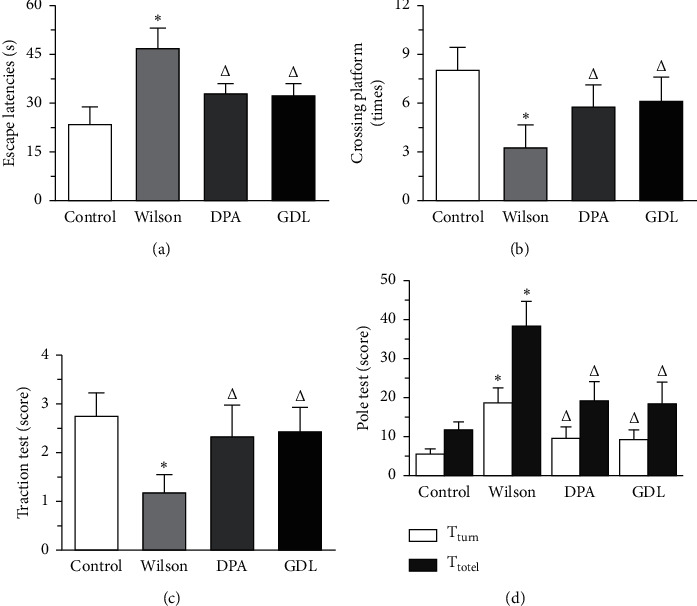
The effects of GDL on behavior in TX mouse model of Wilson disease. (a) The times of escape latencies in all mice. (b) The frequency of crossing the platform in all mice. (c) The scores of the traction test in all mice. (d) The times of pole test in all mice. Data are expressed as the mean ± SD (*n* = 12 per group) and were analyzed by one-way analysis of variance followed by the least significant difference test (Figure 1(a), 1(b), and 1(d)) and Tamhane's T2 test (Figure 1(c)). Control: control group; Wilson: Wilson group; DPA: D-penicillamine group; GDL: Gandouling group; ^*∗*^*p* < 0.01 versus the control group; ^△^*p* < 0.01 versus the Wilson group.

The MWM test outcomes, traction test, and polo test in TX mice with WD are shown in Figures 1(a)–1(d)). Compared with the control group, mice in the Wilson group displayed a significant delay in the times of escape latencies and a remarkable decrease in the frequency across the platform (*p* < 0.01). However, GDL could significantly reduce this delay in escape latencies and increase the frequency of crossing the platform (*p* < 0.01) (Figures 1(a)and 1(b)).

Compared with the control group, mice in the Wilson group displayed a remarkable decrease in the scores of the traction test (*p* < 0.01). However, drug intervention could significantly increase the scores of the traction test (*p* < 0.01) (Figure 1(c)).

Compared with the control group, mice in the Wilson group displayed a remarkable delay in the times of the pole test (*p* < 0.01). However, drug intervention could significantly decrease the delay in the times of pole test (*p* < 0.01) (Figure 1(d)).

As stated above, GDL can significantly improve the behavior of TX mice with WD.

### 3.2. Effects of GDL on Mitochondrial Ultrastructure

**Figure 2:**
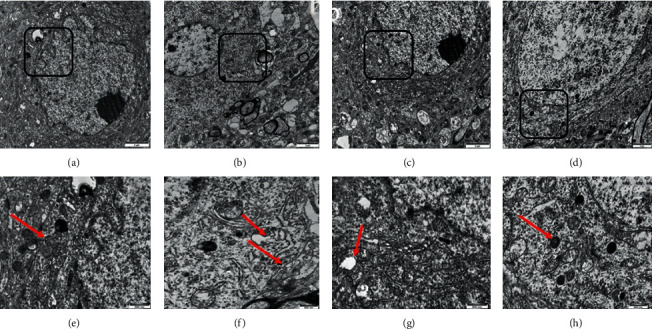
Mitochondrial ultrastructural changes in the hippocampal tissue of mouse model of Wilson disease (2 *μ*m, 500 nm). (a) The control group with normal mitochondrial ultrastructures. (e) Enlargement of the boxed area in (a) and abundant normal mitochondrial ultrastructure were observed. (b) Abundant mitochondria appeared to be dissolved, swollen, show vacuolization, and obvious mitochondrial autophagosomes were observed in the Wilson group. (f) Enlargement of the boxed area in (b). (c) Few mitochondrial vacuolizations were observed in the DPA group. (g) Enlargement of the boxed area in (c). (d) Lysosomes were observed in neurons and no obvious abnormalities were found in the GDL group, and few mitochondria appeared to have vacuolization. (h) Enlargement of the boxed area in (d) and many lysosomes were observed obviously.

Changes in mitochondria ultrastructure are shown in Figure 2. Abundant healthy mitochondria ultrastructure and nuclei were observed in the control group (Figures 2(a) and 2(e)). Mitochondria subject to the Wilson group displayed mitochondrial swelling, cristae decrease or mitochondrial vacuolation, and more obvious mitochondrial autophagosomes (Figures 2(b) and 2(f)). After drug intervention, these types of damage were reduced to a certain degree. Few mitochondrial swelling, vacuolization, or autophagosomes were observed. Some lysosomes were observed in neurons, and no obvious mitochondria ultrastructure abnormalities were founded after GDL treatment (Figures 2(d) and 2(g)). The results demonstrate that GDL could protect the hippocampal neurons against brain injury in TX mice with WD.

### 3.3. Effects of GDL on the Levels of ALT, AST, TBIL, and ALB in TX Mouse Model of WD

**Figure 3:**
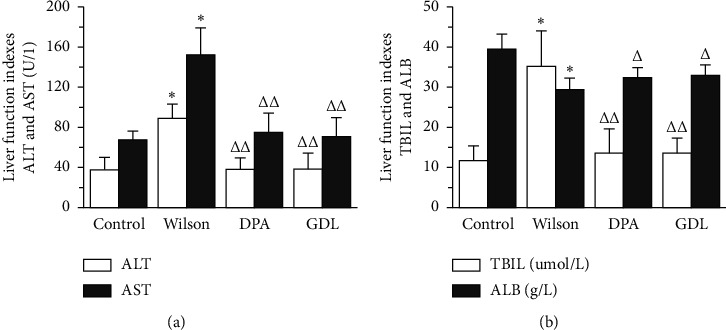
The effects of GDL on the indicators, including ALT, AST, TBIL, and ALB, in TX mouse model of Wilson disease. Data are expressed as the mean ± SD (*n* = 12 per group) and were analyzed by one-way analysis of variance followed by the least significant difference test. (a) The indicators ALT and AST in all mice. (b) The indicators TBIL and ALB in all mice. Control: control group; Wilson: Wilson group; DPA: D-penicillamine group; GDL : Gandouling group; ^*∗*^*p* < 0.01 versus the control group; ^△^*p* < 0.05, ^△△^*p* < 0.01 versus the Wilson group.

The levels of ALT, AST, TBIL, and ALB are shown in Figures 3(a) and 3(b). Compared with the control group, the levels of ALT, AST, TBIL, and ALB were significantly increased in the Wilson group (*p* < 0.01). After drug intervention, these indicators were significantly reduced (*p* < 0.01 or *p* < 0.05). The results demonstrate that GDL can improve the level of liver function in TX mice with WD.

### 3.4. Effects of GDL on Parkin and p62 Colocalization with Mitochondria

**Figure 4:**
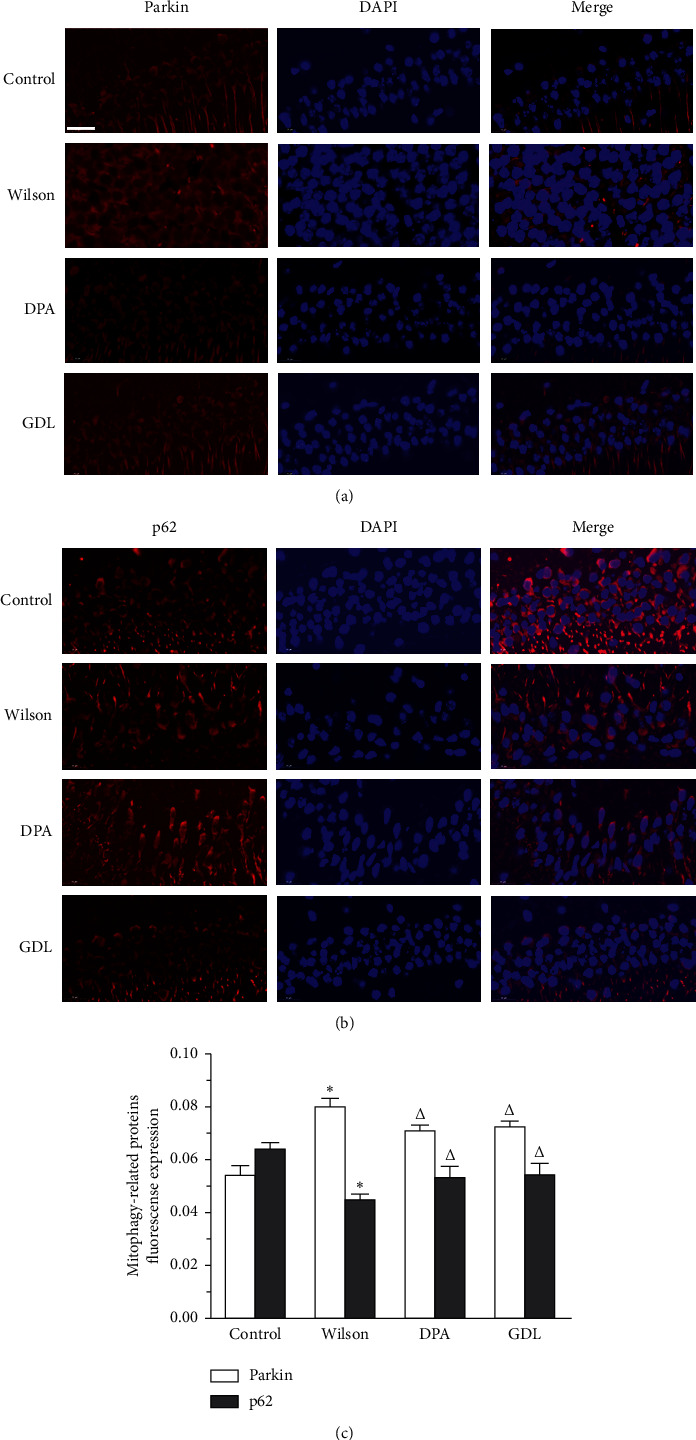
The effects of GDL on parkin and p62 mitophagy-related immunofluorescence expression levels in TX mouse model of Wilson disease (500×, 20 *μ*m). Data are expressed as the mean ± SD (*n* = 3 per group) and were analyzed by one-way analysis of variance followed by the least significant difference test. The blue color is DAPI staining, and the red is neurons. (a) Parkin-positive neurons. (b) p62-positive neurons. (c) Mean fluorescence intensity for parkin and p62. Control: control group; Wilson: Wilson group; DPA: D-penicillamine group; GDL: Gandouling group; DAPI: 4′,6-diamidino-2-phenylindole. ^*∗*^*p* < 0.01 versus the control group; ^△^*p* < 0.05 versus the Wilson group.

The parkin and p62 mitophagy-related immunofluorescence expression levels are shown in Figure 4 (Figures 4(a)–4(c)). Compared with the control group, the expression of parkin was significantly increased in the Wilson group, while p62 was remarkably decreased (*p* < 0.01). After drug intervention, these indicators changed to a certain degree (*p* < 0.05). The results demonstrate that GDL can regulate the mitophagy-related proteins and thus suppress mitophagy in hippocampal neurons.

### 3.5. Effects of GDL on Mitophagy-Related Gene Expression

**Figure 5:**
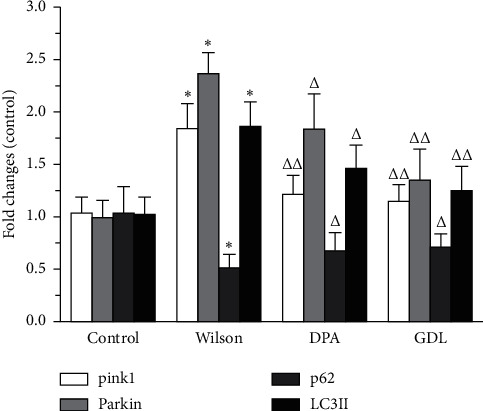
The effects of GDL on pink1, parkin, p62, and LC3II mitophagy-related gene expression levels in TX mouse model of Wilson disease. Data are expressed as the mean ± SD (*n* = 3 per group) and were analyzed by one-way analysis of variance followed by the least significant difference test. Control: control group; Wilson: Wilson group; DPA: D-penicillamine group; GDL: Gandouling group; ^*∗*^*p* < 0.01 versus the control group, ^△^*p* < 0.05, ^△△^*p* < 0.01 versus the Wilson group.

The levels of mitophagy-related gene expression in TX mice with WD are shown in Figure 5. Compared with the control group, the mRNA levels of pink1, parkin, and LC3II expression were significantly increased in the Wilson group, while the level of p62 was remarkably decreased (*p* < 0.01). In addition, GDL intervention could significantly reverse the mRNA levels of pink1, parkin, LC3II, and p62 (*p* < 0.05 or *p* < 0.01).

### 3.6. Effects of GDL on Mitophagy-Related Protein Expression

**Figure 6:**
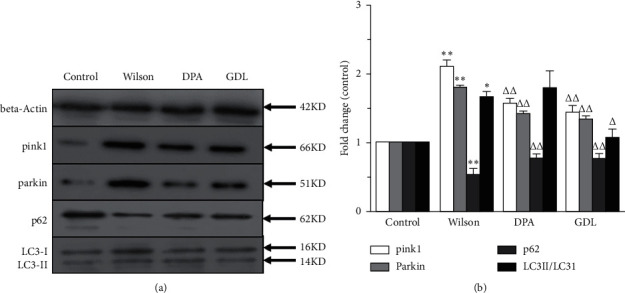
The effects of GDL on pink1, parkin, p62, and LC3 mitophagy-related protein expression levels in TX mouse model of Wilson disease. (a) Western blot assay for pink1, parkin, p62, and LC3. (b) pink1, parkin, p62, and LC3 mitophagy-related protein expression levels with mitochondria. Data are expressed as the mean ± SD (*n* = 3 per group) and were analyzed by one-way analysis of variance followed by the least significant difference test. Control: control group; Wilson: Wilson group; DPA: D-penicillamine group; GDL : Gandouling group; ^*∗*^*p* < 0.05, ^*∗∗*^*p* < 0.01 versus the control group; ^△^*p* < 0.05, ^△△^*p* < 0.01 versus the Wilson group.

The levels of mitophagy-related protein expression in TX mice with WD are shown in Figure 6. Western blot was further conducted to examine the mitophagy-related protein expression levels of pink1, parkin, p62, and LC3II. Compared with the control group, the protein levels of pink1 and parkin, and the rate of LC3II/LC3I expression were significantly increased, and the level of p62 was remarkably decreased in the Wilson group (*p* < 0.05 or *p* < 0.01). GDL could significantly decrease the levels of pink1, parkin, and the rate of LC3II/LC3I, and it increased the level of p62 ((*p* < 0.05 or *p* < 0.01).

As shown above, the regulation of the pink/parkin pathway in TX mice with WD was further confirmed by western blot results to some extent.

## 4. Discussion

In the present study, we found that mitophagy was involved in neurological injury in WD mice and that GDL may inhibit the mitophagy in the hippocampus via regulation of the pink/parkin pathway. GDL treatment significantly inhibited mitophagy activation in the hippocampus, and the reduction of mitophagy may play a vital role in brain injury of WD mice.

GDL is a Chinese patent medicine approved by the National Medical Product Administration, which has been used to treat WD in China for several decades. GDL is composed of *Rheum officinale* Baill (Dahuang), *Coptis chinensis* Franch. (Huanglian), *Scutellaria baicalensis* Georgi (Huangqin), *Curcuma longa* L. (Jianghuang)*, Curcuma rcenyujin* Y *(Yujin), Salvia miltiorrhiza* Bge (Danshen), and *Curcuma phaeocaulis* Valeton (Ezhu). Among these medicines, rhubarb and *Coptis chinensis* are supposed to be the monarch drugs in GDL prescription [22, 23]. Emodin is the main component of rhubarb and has been reported to significantly regulate the expression of mitophagy-related proteins and genes, thereby protecting the damaged mitochondria [29, 30]. On the one hand, damaged mitochondria or excessive degradation can be removed by emodin via the induction of insufficient autophagy or mitophagy. On the other hand, emodin has also been reported to reduce excessive autophagy, and thereby decrease cell apoptosis and brain injury [31–33]. *Coptis chinensis* contains a high dose of zinc, which can chelate with copper and inhibit intestinal absorption of copper [23, 34]. Also, berberine, which is considered to be the main component of *Coptis chinensis*, has been reported to have a protective effect on the brain by reducing the inflammatory response to cerebral ischemia-reperfusion injury [34, 35]. Emerging evidence suggests that berberine contributes to autophagy by up- and downregulation of the levels of key proteins for autophagy [36, 37].

The significant neuroprotective function of GDL in WD has been demonstrated in our previous study [22, 23]. In this study, a significant increase in neurological deficits and dyskinesia was observed in the GDL group, when compared with the control group. All these effects were significantly reversed by GDL intervention. The findings of this study indicated that GDL has a remarkable neuroprotective function in TX mice with WD.

At present, the mechanism of GDL involving in the modulation of neuronal function in the brain is still insufficient. Excessively activated mitophagy in the hippocampus has been reported to aggravate brain damage. With the enhancement of mitophagy, energy metabolism would be affected, mitochondria and macromolecules would be excessively consumed, and this subsequently, results in the enhancement of neuronal apoptosis [38, 39]. Mitophagy has been reported to be involved in the hepatic tissues of WD [17]. However, studies on mitophagy involved in neurons, especially in the brain, are still hard to be detected. In addition, the roles of mitophagy in the neuroprotective mechanism of GDL are unknown, and further investigation is highly warranted. In this study, we found that excessive mitophagy was activated in TX mice with WD. On electron microscopy analysis, it was observed that the swollen and vacuolated mitochondria were shown obviously in the Wilson group. Various degrees of vacuolated mitochondria occurred in the GDL and DPA groups, but not as serious as that in the Wilson group. These electron microscopy results indicated that GDL treatment can inhibit mitophagy and protect neurons in the hippocampus in WD.

The pink1/parkin-dependent mitophagy pathway plays a critical role in the autophagic removal of damaged mitochondria, and it has also been reported to be involved in WD [17, 40]. Emerging evidence suggests that parkin contributes to mitophagy by specifically targeting depolarized mitochondria [40]. Pink1 acts as an upstream factor for parkin, which is essential for both activation of parkin activity and recruitment of parkin onto depolarized mitochondria [41–43]. In healthy mitochondria, pink1 is continuously transferred to the mitochondrial intima, and it is subsequently cleaved by PALP (presenilin-associated rhomboid-like protein) [44]. Therefore, under healthy conditions, pink1 maintains a low expression level and is hardly detected. When mitochondria are severely damaged, their membrane potential is reduced, resulting in the blockage of transport of pink1. Excessive accumulation of pink1 on mitochondrial outer membrane recruits parkin from the cytosol to the mitochondria outer membrane. Then, parkin connects the receptor protein SQSTM1 (ubiquitin-binding adapter: p62) to the mitochondria by ubiquitination of various substrates. Subsequently, after the bond of the phagocytic membrane surface protein LC3, the autophagic clearance of mitochondria is initiated by lysosomes [45, 46]. LC3 is present as LC3-I (cytoplasmic form) and LC3-II (processed form), and it is the only protein marker reliably associated with mature autophagosomes. Currently, detecting LC3II/LC3-I has become a reliable method for monitoring autophagy and autophagy-related processes, including autophagic cell death [47].

In the present study, the immunofluorescence labeling technique was used to detect the colocalization of parkin and p62 and to subsequently analyze the changes in the pink1/parkin-dependent mitophagy pathway in the hippocampus. From images and colocalization analysis, it was observed that colocalization of parkin with mitochondria was significantly decreased, while p62 was remarkably increased with GDL treatment. These results indicated that GDL intervention had a remarkable effect on parkin and p62 colocalization. Moreover, GDL intervention had a significant inhibitory effect on parkin, indicating that GDL may improve neurological functions by the inhibition of the pink1/parkin-dependent mitophagy pathway.

The mRNA and protein levels of pink1, parkin, p62, and LC3II in the hippocampal tissues were detected. Compared with the control group, the mRNA levels of pink1, parkin, and LC3II were significantly increased, while the p62 level was significantly decreased in the Wilson group. Also, the protein levels of pink1, parkin, and the rate of LC3II/LC3I significantly increased, while the level of p62 significantly increased in the Wilson group.

It was observed that the genes and proteins levels of pink1, parkin, and LC3II (or the rate of LC3II/LC3I) were significantly decreased, while the p62 level was remarkably increased in the GDL group, which further indicated that the neuroprotective mechanism of GDL may occur via the inhibition of excessive mitophagy by the regulation of pink1/parkin pathway in TX mouse brain of WD.

Our previous studies have already confirmed that GDL has a remarkable protective effect on liver injury [22, 23]. In this study, the hepatic function was significantly improved after the GDL intervention. The indicators, including ALT, AST, TBIL, and ALB, were obviously improved in the drugs' therapy group during the 8-week treatment, when compared to mice in the Wilson group, which further indicates that GDL has a remarkable protective effect in liver injury.

In conclusion, the present study demonstrated that mitophagy was increased in the hippocampal tissues of TX model mice in WD. The protein levels of pink1, parkin, and LC3II (or the rate of LC3II/LC3I) were increased, while the p62 level was decreased in the TX mouse model of WD. Additionally, inhibition of the pink1/parkin-dependent mitophagy pathway is believed to be the main neuroprotective mechanism for GDL in WD. Drug interventions, especially GDL, could improve neurological syndromes, attenuate and suppress mitophagy, and correct the transcriptional levels and protein expression by the regulation of the pink1/parkin signaling pathway. However, whether GDL can regulate the other proteins or pathways, such as the P13k/Akt/mTOR, AMPK/mTOR/ULK1, or Wnt/catenin pathway, to inhibit autophagy needs to be explored. Further investigation is warranted to elucidate the specific potential mechanisms.

## Data Availability

The data used to support the ﬁndings of this study are available from the corresponding author upon request.
